# Polymer Nanodiscs: Discoidal Amphiphilic Block Copolymer Membranes as a New Platform for Membrane Proteins

**DOI:** 10.1038/s41598-017-15151-9

**Published:** 2017-11-09

**Authors:** Mariana C. Fiori, Yunjiang Jiang, Wan Zheng, Miguel Anzaldua, Mario J. Borgnia, Guillermo A. Altenberg, Hongjun Liang

**Affiliations:** 1 Department of Cell Physiology and Molecular Biophysics, and Center for Membrane Protein Research, Texas Tech University Health Sciences Center, Lubbock, Texas USA; 2 Department of Chemical Engineering, Texas Tech University, Lubbock, Texas USA; 3 Laboratory of Cell Biology, Center for Cancer Research, National Cancer Institute, National Institutes of Health, Bethesda, Maryland USA

## Abstract

Lipid nanodiscs are playing increasingly important roles in studies of the structure and function of membrane proteins. Development of lipid nanodiscs as a membrane-protein-supporting platform, or a drug targeting and delivery vehicle in general, is undermined by the fluidic and labile nature of lipid bilayers. Here, we report the discovery of polymer nanodiscs, *i.e*., discoidal amphiphilic block copolymer membrane patches encased within membrane scaffold proteins, as a novel two-dimensional nanomembrane that maintains the advantages of lipid nanodiscs while addressing their weaknesses. Using MsbA, a bacterial ATP-binding cassette transporter as a membrane protein prototype, we show that the protein can be reconstituted into the polymer nanodiscs in an active state. As with lipid nanodiscs, reconstitution of detergent-solubilized MsbA into the polymer nanodiscs significantly enhances its activity. In contrast to lipid nanodiscs that undergo time- and temperature-dependent structural changes, the polymer nanodiscs experience negligible structural evolution under similar environmental stresses, revealing a critically important property for the development of nanodisc-based characterization methodologies or biotechnologies. We expect that the higher mechanical and chemical stability of block copolymer membranes and their chemical versatility for adaptation will open new opportunities for applications built upon diverse membrane protein functions, or involved with drug targeting and delivery.

## Introduction

Membrane proteins (MPs) are encoded by 20 to 30% of the sequenced genomes, and are the targets of most pharmacological agents^1,2^. Mutations of MPs are associated with many disorders, including cystic fibrosis, cerebrovascular accidents, deafness, cardiac infarcts, and neurodegenerative diseases^3–7^. Understanding the structure and function of MPs is of great importance and frequently requires work with purified MPs reconstituted in a model membrane. Ideally, this membrane platform should mimic native biomembranes to maintain the structural and functional integrity of MPs, and be robust and reliable under a broad range of abiotic conditions in long term for methodology and technology development.

Liposomes are a popular MP-supporting platform, but the fluidic and labile nature of lipid bilayers limits their utility^8–13^. In some cases, the secluded intraliposomal side constitutes a challenge for studies that involve ligand binding. The relatively large size of liposomes also complicates optical spectroscopy measurements due to light scattering. Lipid nanodiscs (LNDs) have emerged as a MP-supporting platform that overcomes some of the limitations of liposomes^14–17^. LNDs represent a small discoidal lipid bilayer patch encased within two belt-like membrane scaffold proteins (MSPs) derived from apolipoprotein A1, a major component of serum high-density lipoprotein complexes^17,18^. The diameter of LNDs ranges from 8 to 16 nm, depending largely on the length of the MSPs. This size range displays sufficient flexibility to accommodate a variety of MPs^14–17^. Their homogeneous and monodisperse nature, ready accessibility to both extramembrane sides of the reconstituted MPs, and low light scattering are some of the prominent advantages of LNDs for methodologies such as luminescence spectroscopy, solution NMR spectroscopy and single-particle cryo-electron microscopy (cryo-EM)^15,19–22^. LNDs have also gained increasing interests as a drug targeting and delivery platform^23–25^. Despite the promise and progress empowered by LNDs, the inherent instability of lipid bilayers and their inescapable structural evolution during storage and shipment is problematic for the development of LND-based diagnostic and therapeutic products. Crosslinking, bonding with supporting substrates and encapsulation have been exploited to improve the stability of lipid bilayers^10–13^, but these modifications will likely compromise the structure and function of the embedded MPs.

**Figure 1:**
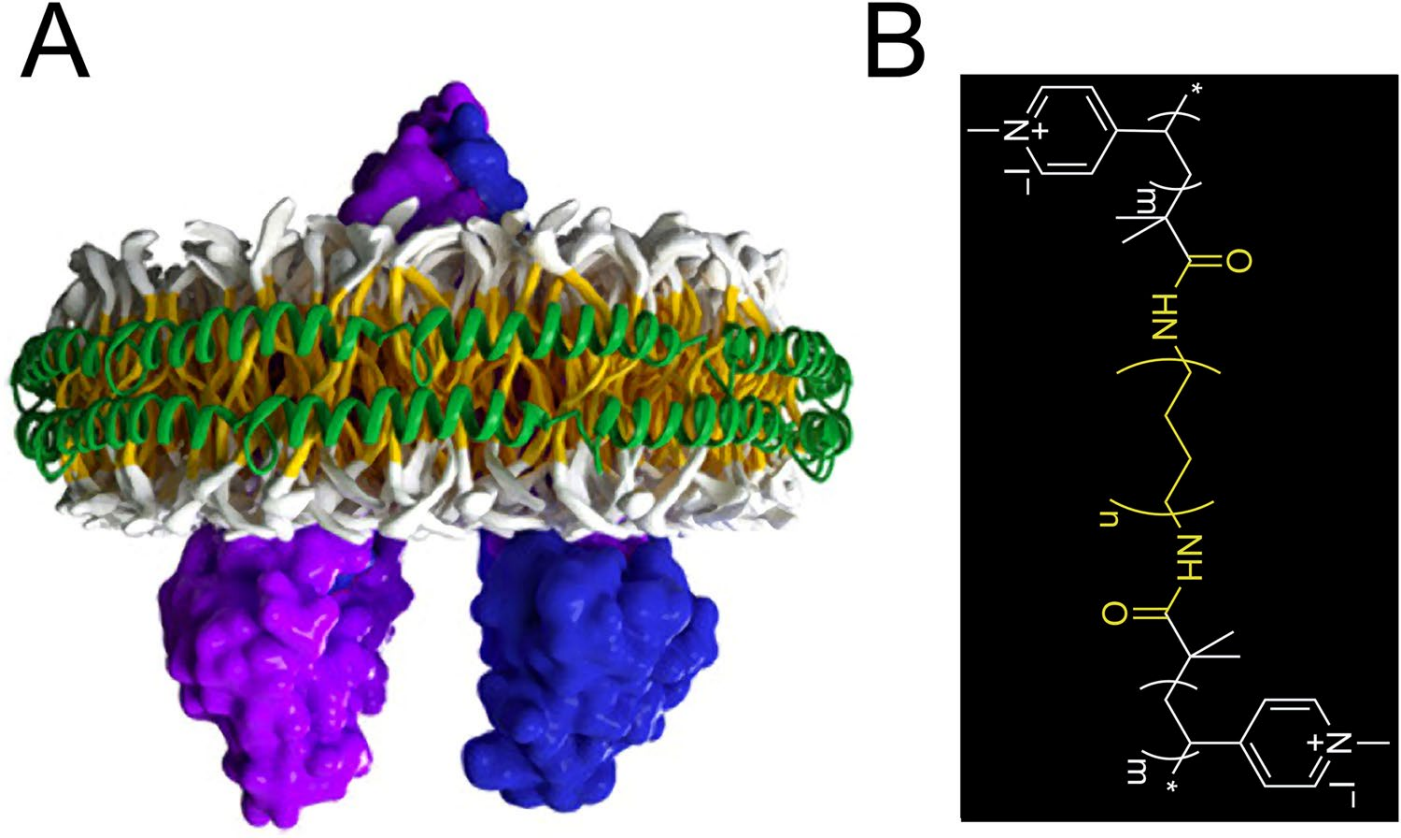
Polymer nanodiscs. (**A**) Illustration of a PND with reconstituted MsbA. The amphiphilic block copolymer membrane patch comprised of hydrophobic membrane-forming (gold) and hydrophilic membrane-surface blocks (gray) is encased within two MSPs (green ribbon coils). One reconstituted MsbA dimer (subunits in purple and blue) is also shown. This PND model is derived from our spectroscopy and microscopy analysis, and available information on the LND structure. (**B**) Chemical structure of the amphiphilic block copolymer HPBD-*b*-(P4MVP_28_)_2_. The hydrophobic HPBD block (*i.e*., hydrogenated 1,4-polybutadiene) and the hydrophilic P4MVP blocks (*i.e*., poly(4-vinyl-N-methylpyridine iodide) are shown in yellow and white, respectively. The letters m and n signify the number of repeating units. See Supplementary Information for details.

Here, we report the discovery of polymer nanodiscs (PNDs) consisting of discoidal amphiphilic block copolymer membrane patches encased within MSPs as a new platform of improved stability that supports MPs (Fig. 1A). Amphiphilic block copolymers can self-assemble in water spontaneously to form polymersomes, *i.e*., liposome-like polymer vesicles^26,27^. In contrast to lipids, block copolymers have low critical micelle concentrations (CMCs) and a much-enhanced chemical and mechanical stability, as well as practically unlimited choices of chemical variations on individual repeating units. These advantages have prompted many explorative studies to adapt polymersomes as liposome-substitutes to support MPs^8,28–35^, or to deliver pharmaceuticals^36,37^. We demonstrate here, that in the presence of detergents and MSPs, selective transition from polymersomes to PNDs occurs upon detergent removal, underlying a broadly applicable physical principle that guides the transition from vesicle to nanodisc^38,39^. Using the bacterial ATP-binding cassette (ABC) transporter MsbA^40–42^ as a MP prototype, we show that reconstitution of an individual MsbA dimer (functional unit) in PNDs is possible, and this reconstitution significantly improved the activity of MsbA compared to its detergent-solubilized form. In contrast to LNDs that experience time- and temperature-dependent aggregation, the chemically and mechanically more stable PNDs show negligible structural change upon storage at 4 °C, 20 °C or 37 °C. This study illuminates the potential of PNDs as a new MP-supporting platform with enhanced stability, which is critical for the development of nanodisc-based characterization methodologies, and for diagnostic or therapeutic applications.

## Results and Discussion

### Support of MPs in amphiphilic block copolymer membranes

While the roles of specific endogenous lipids on the function of some MPs have been established^43–46^, it has been recognized that a major role of lipids is related to their contribution to the bulk physicochemical properties of biomembranes, such as curvature, lateral pressure profile, and thickness^47–51^. In this context, it is not surprising to observe that a large number of detergent-solubilized MPs retain their functions after reconstitution in lipid bilayers of very simple compositions, suggesting that the specific endogenous lipids critical for these MPs, if exist, may be bound tightly to the MPs and survive the detergent solubilization and reconstitution steps^44,52–54^. The non-exclusive partnership between MPs and native biomembranes opens up opportunities to study the structure and function of MPs in simple model membranes, including synthetic polymer membranes that mimic the physicochemical properties of biomembranes^8,28–35^, and to develop MP-based biotechnology^9,55,56^. It should be noted that endogenous lipids can be still doped into the synthetic membranes when needed^57,58^. Polydimethylsiloxane (PDMS)-based triblock copolymers membranes have been used to support MPs such as OmpF, aquaporin, ATP synthase, and a potassium channel^31–35^. Due to the low glass transition temperature of PDMS, these membranes are in a viscous fluid state at room temperature, which is undesirable for many biotechnological applications. To address the membrane stability issue and to understand the role of membranes on defining MP functions, we developed polybutadiene (PBD)- and polystyrene (PS)-based block copolymer membranes of increased membrane stability^8,28–30^. We showed that functional reconstitution of proteorhodopsin, a light-driven proton pump^8,28^, bacterial reaction center, a light-driven electron-hole generator^29^, and bovine rhodopsin, a canonical prototype of G-protein coupled receptors (GPCRs)^30^ into these membranes is possible. While the reaction center-mediated electron-transport kinetics appear insensitive to different membranes^29^, the proton-pumping photocycle of proteorhodopsin is allosterically slowed down as the membrane flexibility decreases^8^. Even glassy PS membranes with superior bulk-state stability can be tuned to bear sufficient chain-motion freedom at the nanoscale to rival lipid bilayers for supporting the conformational changes of proteorhodopsin, underscoring the versatility of polymer membranes to support MPs with optimized stability and performance^8^. The versatility was also demonstrated by the discovery of a new activation mode for bovine rhodopsin: we revealed that the attractive charge interaction between the polymer membrane surface and the deprotonated Glu134 residue of the rhodopsin-conserved ERY sequence motif can be introduced to replace the role of native biomembranes in breaking the cytoplasmic “ionic lock” of rhodopsin to dock transducin^30^.

### Synthesis and characterization of an amphiphilic block copolymer membrane

**Figure 2:**
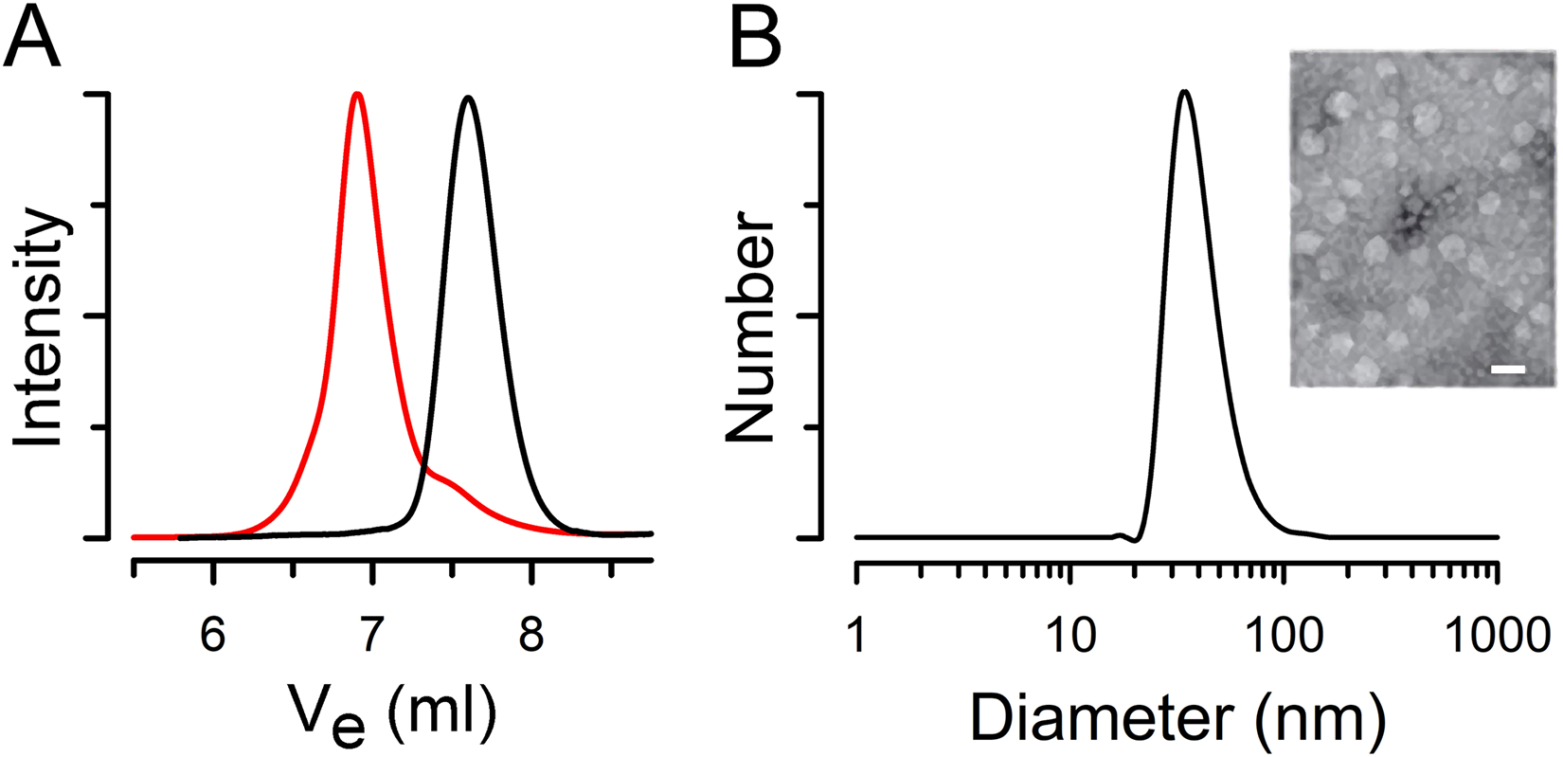
Well-defined amphiphilic block copolymer HPBD-*b*-(P4MVP_28_)_2_ self-assembles in water to form polymersomes. (**A**) SEC traces of HPBD (black; Mn = 2,230 Da, PDI = 1.14) and HPBD-*b*-(P4VP_28_)_2_ (red; Mn = 8,690 Da, PDI = 1.16). Ve: elution volume. (**B**) DLS of self-assembled HPBD-*b*-(P4MVP_28_)_2_ in water showing the formation of polymersomes of different sizes (average of 41-nm diameter). Number: number of particles. The polymersomes can be directly observed under TEM (inset; scale bar: 50 nm).

As a model system to test the feasibility of producing PNDs, we used the hydroxyl-terminated, hydrogenated polybutadiene (HPBD-(OH)_2_) that is commercially available (Krasol® HLBH-P 2000 from Cray Valley USA) as the building block to prepare the well-defined triblock copolymer HPBD-*b*-(poly(4-vinylpyridine)_28_)_2_ (HPBD-*b*-(P4VP_28_)_2_) *via* reversible addition-fragmentation chain transfer (RAFT) polymerization. The HPBD is more stable than PBD due to the lack of unsaturated bonds, and its hydroxyl end groups were further converted to amines in order to form amide bonds with the P4VP blocks instead of the more labile ester bonds. We then used the quaternization reaction to convert the P4VP blocks into hydrophilic poly(4-vinyl-N-methylpyridine iodide) (P4MVP). The reaction scheme to prepare the amphiphilic triblock copolymer HPBD-*b*-(P4MVP_28_)_2_ and its structural characterization are shown in the Supporting Information. Both nuclear magnetic resonance (NMR) spectroscopy (Supplementary Figs 3–4) and size-exclusion chromatography (SEC) analysis (Fig. 2A) confirmed the successful synthesis of well-defined HPBD-*b*-(P4VP_28_)_2_ (Fig. 1B) with a small polydispersity index (PDI). The amphiphilic HPBD-*b*-(P4MVP_28_)_2_ self-assembles spontaneously in water to form polymersomes of different sizes, with an average diameter of ~40 nm as determined by the number distribution of dynamic light scattering (DLS) data (Fig. 2B; see Supplementary Fig. 5 for the intensity distribution).

### PNDs as a new membrane platform for MPs

To take advantage of the nanodisc platform^14–17,23–25^ while addressing the fluidic and labile nature of lipid bilayers, we proposed the concept of PND and hypothesized that the transition from polymersomes to PNDs upon removal of detergent from a detergent-MSP-amphiphilic block copolymer mixture shares similar driving forces to those involved in the formation of LNDs. Inspired by the formation mechanism and pathway for LNDs^14–17,38,39^, we successfully developed a relatively simple and efficient protocol (see details in Materials and Methods) starting with a mixture of MSP1E3D1 and detergent-solubilized HPBD-*b*-(P4MVP_28_)_2_, followed by dilution of the detergent, and purification of PNDs by immobilized metal-affinity chromatography (IMAC) based on the affinity of the MSP poly-His tag for Ni^2+^.

**Figure 3:**
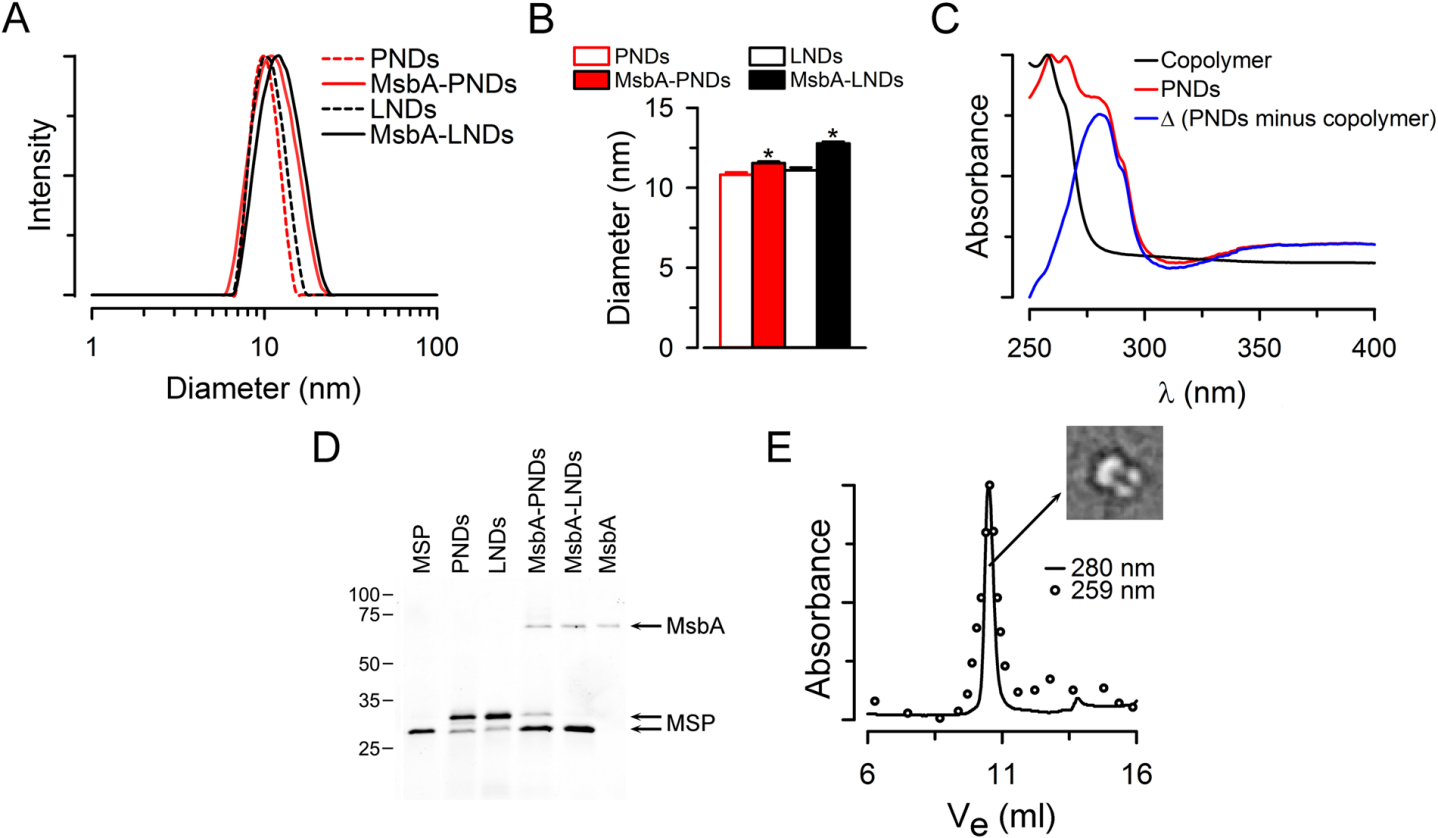
Characterization of PNDs and their comparison with LNDs. (**A**) Typical examples illustrating PNDs and LNDs hydrodynamic diameter distributions determined by DLS. (**B**) Summary of the average hydrodynamic diameter data showing means ± SEM of PNDs (n = 10), MsbA-PNDs (n = 7), LNDs (n = 7) and MsbA-LNDs (n = 7). The asterisks denote P < 0.002 *vs*. the corresponding MsbA-loaded nanodiscs. (**C**) Absorption spectra of PNDs and the amphiphilic block copolymer itself in solution. Spectra are normalized to the corresponding peak values. The difference between the spectra is shown in blue. (**D**) Samples of a representative gel (16% SDS-PAGE) stained with Instant Blue for protein detection. Samples are indicated on top of the lanes. MsbA refers to MsbA T561C and MSP to MSP1E3D1 (equal amounts in moles). The 2 MSP arrows point to MSP1E3D1 with (top) and without (bottom) cleavage of the poly-His tag. MSP in lane 1 and MsbA in lane 6 correspond to purified MSP1E3D1 and MsbA (DDM-solubilized MsbA T561C), respectively. The positions of molecular mass markers (in kDa) are indicated on the left. The lanes are from the same gel, but the MSP, PNDs/LNDs and MsbA-PNDs/MsbA-LNDs/MsbA lanes were not adjacent (original gel presented as Supplementary Fig. 7). (**E**) Typical SEC of PNDs revealing the co-existence of MsbA and the block copolymer membrane. The sample was run on a PL Aquagel-OH 50 column SEC column (see Materials and Methods for details). The absorbance at 280 nm (line) was used to detect MSP and MsbA tryptophans, and the absorbance at 259 nm (circles) was used to follow the block copolymer membrane. Note that the block copolymer has an absorbance peak at 259 nm due to its pyridine moieties but no absorbance at 280 nm (panel C). Ve: elution volume. Inset: an example of the TEM of MsbA-loaded PND that resembles MsbA in LNDs^66^ and our PND illustration (Fig. 1A).

DLS studies (Fig. 3A) of the PNDs (dotted red trace) revealed fairly monodispersed nanoparticles with an average diameter of ~11 nm and a size distribution similar to that of LNDs (dotted black trace) prepared with the same MSP and *E*. *coli* polar lipid extract. The average data presented in Fig. 3B confirmed the similar sizes of PNDs (empty red bar) and LNDs (empty black bar). Also, the polydispersity index (PDI) calculated from the DLS data was similar for PNDs (8 ± 1%, n = 10) and LNDs (6 ± 1%, n = 7). Figure 3C shows that the PNDs contain both copolymer and MSPs. The differential absorption spectra of empty PNDs (red) and the block copolymer itself in solution (black) clearly shows the tryptophan absorbance (blue) corresponding to the MSPs. The presence of MSPs in the PNDs was also illustrated by the MSPs in gels from PNDs stained for protein detection (Fig. 3D). Taken together, these data indicate that PNDs of fairly uniform size that contain MSPs and the copolymer can be produced using our protocol.

To test the reconstitution of MPs in PNDs we used the active mutant T561C of MsbA, a frequently used bacterial model for structural and functional studies of ABC exporters^22,40–42^. MsbA is a flippase that translocates lipid A, an endotoxin component, from the inner to the outer leaflet of the inner membrane of Gram-negative bacteria^22,42^. To produce MsbA-loaded PNDs we followed the protocol used for the formation of empty PNDs, but added detergent-solubilized MsbA to the MSP1E3D1/block copolymer mixture, and used a MSP1E3D1 without the poly-His tag. The tag of this MSP was removed by cleavage by TEV protease, and the untagged MSP was isolated as the flow through of an IMAC column. By using MsbA with a poly-His tag and untagged MSP we could easily separate by IMAC the MsbA-loaded PNDs from empty ones. The incorporation of MsbA in the PNDs was confirmed by the increased hydrodynamic diameter determined by DLS (Fig. 3A, solid red *vs*. dotted red traces; and Fig. 3B, solid red *vs*. empty red bars), the presence of MsbA and MSP in gels of PNDs stained for protein detection (Fig. 3D), and the co-localization of protein (absorbance at 280 nm; A_280_) and copolymer (absorbance at 259 nm; A_259_) in high-resolution size-exclusion chromatograms (Fig. 3E). Using cryo-EM, we also directly observed the MsbA-carrying PNDs (Fig. 3E, inset). As revealed by the gel in Fig. 3D, the proportion of MSP1E3D1 to MsbA was similar in PNDs (2.2 ± 0.1; n = 11) and LNDs (1.8 ± 0.1; n = 5), strongly suggesting that individual PNDs are encased within two copies of MSPs just like LNDs, as depicted in the PND model (Fig. 1A). Since Instant Blue stains MSP1E3D1 more than MsbA (see Fig. 1A), for the calculation of the MSP1E3D1/MsbA ratios above we used purified proteins whose concentration were determined by their A_280_ and extinction coefficients.

### PNDs are a more robust and reliable MP-supporting platform than LNDs

**Figure 4:**
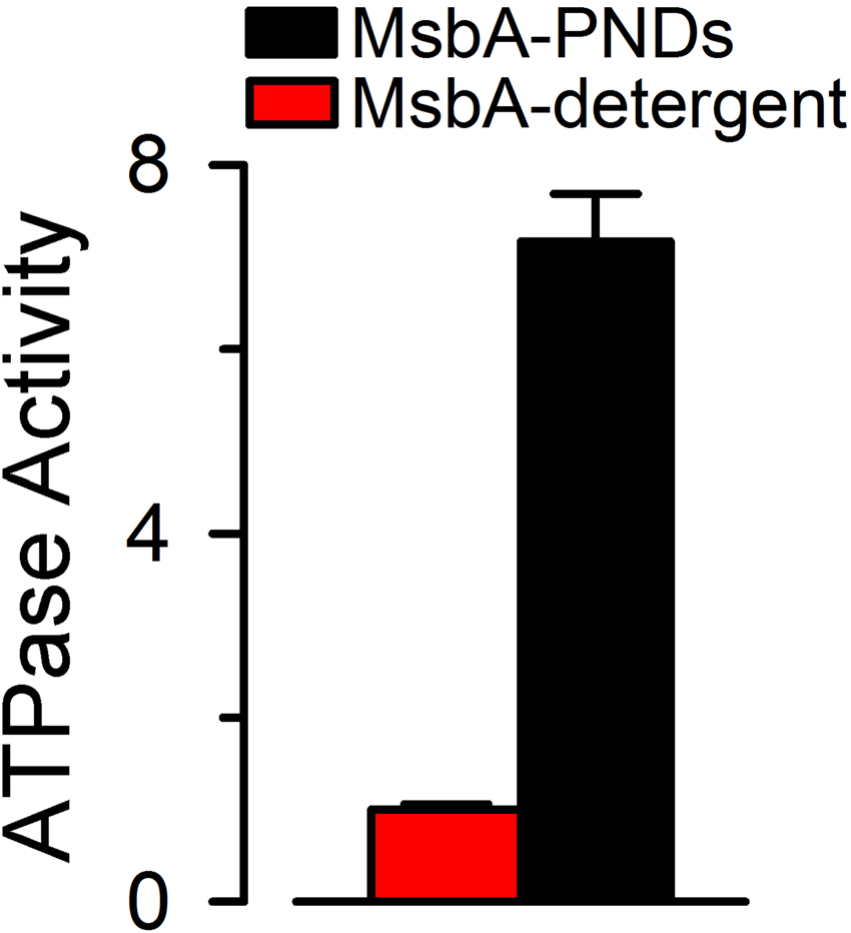
ATPase activity of MsbA reconstituted in PNDs. The ATPase activity of purified MsbA T561C was measured at 37 °C. Values are presented as means ± SEM relative to the activity in detergent (0.35 ± 0.02 s^−1^). The activity of MsbA in PNDs (n = 12) was significantly higher (~7×) than that of MsbA in detergent (n = 10; P < 0.001).

Although the PNDs membrane is made entirely by synthetic block copolymers without biological lipids, MsbA reconstituted in PNDs displays an ATPase activity several folds higher (~7×) than that of the detergent-solubilized MsbA (Fig. 4). This enhanced activity is similar to that reported previously for MsbA reconstituted in LNDs comprised of *E*. *coli* polar lipid extract (~9× the value in detergent)^22^. Since no additional endogenous lipids were added during the reconstitution of detergent-solubilized MsbA into the PNDs, our findings suggest that the physicochemical properties rather than specific chemical compositions of the membrane play an important role in supporting MsbA activity, and that it is possible to reconstitute functional MPs in PNDs. Nanodiscs display free accessibility to both sides of the reconstituted MPs. Whereas this is advantageous for many applications, it limits functional assessment of transport proteins because transport assays cannot be performed with both sides exposed to the same solution. In the particular case of MsbA, we can measure ATPase activity, but not transport. Therefore, when we say active MsbA we refer to MsbA with ATPase activity because functional reconstitution (substrate transport) cannot be assayed.

**Figure 5:**
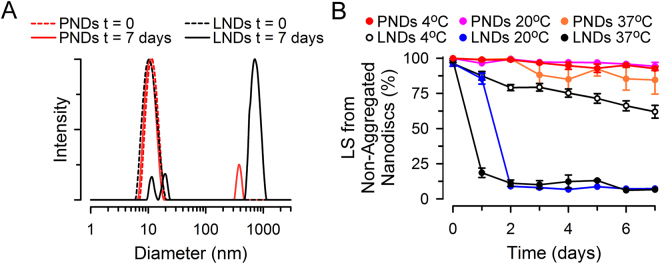
Stability of PNDs. (**A**) Typical examples illustrating hydrodynamic diameter distributions determined by DLS of PNDs and LNDs incubated at 37 °C. (**B**) Aggregation of PNDs and LNDs kept for 1 week at 4 °C, 20 °C or 37 °C, as revealed by DLS. The figure shows the percentage of light scattered (LS) by nanodiscs consisting of a monodisperse population of ~11-nm diameter assessed from size-intensity distributions such as those shown in panel A. See Materials and Methods for details. Data are means ± SEM of 6–7 independent experiments in all conditions, except for PNDs and LNDs at t = 0 (n = 13 and 11, respectively), and PNDs at 20 °C in days 6 and 7, where n = 1 and 2, respectively, and the single measurement and average are reported. SEMs smaller than the symbols are not shown. Differences between PNDs and LNDs were statistically significant (P < 0.001) from day 1 at 37 °C and day 2 onwards at 4 and 20 °C.

Given the fairly low gel-to-liquid transition temperature and fairly high CMC of many lipids, it has been recognized that the stability of LNDs depends highly on their lipid composition^59–61^. The instability of liposomes and LNDs is not particularly troublesome in research labs, as we generally prepare and test freshly made samples under well-controlled conditions (*e.g*., at 20 °C with short-term storage at 4 °C). However, it could become a prohibitive challenge for applications under a broad range of harsher, abiotic conditions, such as those needed for remote collaborations that require sample exchange, or for the development of MP-based diagnostic and therapeutic products. When we analyzed LNDs, we observed their aggregation over time by light scattering measurements. Light scattering is very sensitive to aggregation because of the steep dependency of scattering intensity on particle size, and therefore, intensity distributions overestimate the percentage of aggregated particles. In Fig. 5 we present light scattering intensity data because they are our primary measurement and are the most sensitive to aggregation, but examples of particle number distributions of the data can be found in Supplementary Fig. 6. The examples of LNDs in Fig. 5A illustrate that after 7 days of storage at room temperature most of the LND sample's scattered light comes from large aggregated particles (1,000 to 2,000-nm diameter; solid black trace) rather than particles similar to “fresh” LNDs of ~11-nm diameter (dotted black trace). In contrast, for PNDs stored under the same conditions, after 7 days most of the scattered light still comes from non-aggregated PNDs (solid red trace) that have the same size as “fresh” PNDs (~11-nm diameter; dotted red trace). The average data of multiple measurements quantitatively supporting increased stability of PNDs are depicted in Fig. 5B, which shows the percentage of scattered light coming from non-aggregated nanodiscs as a function of storage time and temperature. The LNDs show rapid aggregation at 37 °C (black circles), and in just 2 days when stored at 20 °C (blue circles). This aggregation behavior is alleviated when stored at 4 °C (open circles), but is still significant. In contrast, the PNDs do not show aggregation when stored at 4 °C, 20 °C or 37 °C for 1 week. The aggregation of LNDs does not seem to be related to the use of *E*. *coli* lipids because in pilot experiments LNDs with bilayers formed by 1,2-dimyristoyl-*sn*-glycero-3-phosphocholine (DMPC) also showed instability; only ~15 and 5% of the scattering signal came from the ~11-nm DMPC nanodiscs stored for 4 days at 4 °C and 20 °C, respectively. MsbA-PNDs were also more stable than MsbA-LNDs. After storage for 6 days at 4 °C, 93 ± 2% of the scattered light still comes from non-aggregated MsbA-PNDs (n = 4), whereas the value for MsbA-LNDs was 79 ± 1% (n = 3; P < 0.003 *vs*. MsbA-PNDs). Measurements after freezing at -80 °C and thawing on ice showed 99 ± 1 (n = 4) and 96 ± 1% (n = 3) of the scattered light arising from non-aggregated PNDs and MsbA-PNDs, whereas significant decreases were observed for LNDs (37 ± 4%; n = 4; P < 0.001) and MsbA-LNDs (29 ± 9%, n = 4; P < 0.03). The ATPase activity of MsbA in PNDs was not affected by freezing and thawing (Δ = +13 ± 15%; n = 4 from 2 independent MsbA-PND preparations). Overall, these results show that PNDs constitute a more robust platform than LNDs.

## Conclusions

In summary, we developed the concept and protocol to prepare PNDs comprised of well-defined amphiphilic block copolymer membranes to address the inherent limitation of LNDs. Using MsbA as a MP prototype, we demonstrated that reconstitution of detergent-solubilized MsbA in the PNDs increases its activity, similar to that observed for the reconstitution of MsbA in LNDs. An important difference between PNDs and LNDs lies in their stability: unlike LNDs that aggregate significantly in a short time, PNDs show negligible time- and temperature-dependent structural evolution. PNDs are therefore better suited for applications that need survival in a broad range of abiotic conditions in long term. Due to the higher mechanical and chemical stability of block copolymer membranes and their chemical versatility for adaptation, such as variation of membrane thickness and moduli, interfacing with supporting substrates, adding labels or specific recognition groups, to name a few, and the advantages of nanodiscs over other membrane platforms, the development of PNDs could have a powerful impact on biotechnology and biomedical applications built upon diverse MP functions or involved with drug targeting and delivery.

## Methods

### Expression and purification of the MSP1E3D1

MSP1E3D1 was expressed in the *E*. *coli* strain BL21 DE3-RILP (Agilent Technologies) transformed with the plasmid pMSP1E3D1 (Addgene). Expression was induced at OD_600_ ~1 with 1 mM isopropyl-β-D-thiogalactopyranoside and the cells were harvested after growing for 2 h at 37 °C. MSP1E3D1 was purified by IMAC using Ni-NTA agarose beads (Qiagen), as described previously^14,22^. For some experiments, the poly-His tag of the MSP was removed by digestion with TEV protease and the non-tagged MSP was isolated as the flow through from a column packed with Ni-NTA^22,62^. Protein concentration was determined from the absorbance at 280 nm (A_280_) and purity was estimated at > 95% from SDS-PAGE gels stained with Instant Blue (Expedeon).

### MsbA expression, purification, and activity assay

MsbA T561C (an active mutant that we have studied extensively)^22,42^ was expressed and purified as previously described^22,42^. Briefly, MsbA T561C expressed in BL21 DE3-RILP *E*. *coli* was solubilized from membranes with *n*-dodecyl-β-D-maltopyranoside (DDM; Inalco Pharmaceuticals), and purified by IMAC (Talon Superflow; Clontech) followed by SEC using a Bio-scale Mini Bio-Gel P-6 DC (Bio-Rad Laboratories) equilibrated with 100 mM NaCl, 20 mM Tris/HCl, pH 7.5, with 0.065% DDM, 0.04% sodium cholate, 15% glycerol and 0.2 mM TCEP. Purified MsbA T561C was stored at −80 °C until use. As for MSP, protein concentration was determined from the A_280_ and purity was estimated at > 95% from SDS-PAGE gels stained with Instant Blue. ATPase activity was measured as described^22,42,63^, using a variant of the ATPase linked assay. DDM at the concentration used does not interfere with the assay.

### Production of lipid nanodiscs (LNDs)

LNDs were assembled following a published protocol^14,22^. *E*. *coli* polar lipid extract in chloroform (Avanti Polar Lipids) was dried overnight, reconstituted in nanodisc buffer (100 mM NaCl, 20 mM Tris/HCl, pH 7.5, 0.1 mM TCEP) with 100 mM sodium cholate and sonicated for several minutes. For the formation of LNDs we used an MSP:lipid molar ratio of 1:100, and for the MsbA-loaded LNDs (MsbA-LNDs) we used an MsbA:MSP molar ratio of 1:6. The mix was incubated for 1 h at 4 °C with gently rotation, and the self-assembly process was initiated upon detergent removal by incubation at 4 °C overnight with Biobeads SM-2 (Bio-Rad Laboratories). The LNDs were purified by SEC using a Superdex 200 Increase 10/300 column (GE Healthcare) equilibrated in nanodisc buffer, with a flow of 0.5 ml/min, and collection of 1-ml fractions for isolation of relevant peaks used in the studies. MsbA and MSP concentrations in the LNDs samples were estimated in SDS-PAGE gels stained with Instant Blue, using known amounts of purified MsbA and MSP as standards.

### Production of polymer nanodiscs (PNDs)

The amphiphilic HPBD-*b*-(P4MVP_28_)_2_ triblock copolymer was dissolved in 100 mM NaCl, 20 mM Tris/HCl, pH 7.5, with 80 mM *n*-octyl-β-D-glucopyranoside (OG; Anatrace) to a final concentration of 40 mg/ml (~2.5 mM). The solution was sonicated 3 times for 10 min each, and was flash-frozen in liquid N_2_ and thawed on ice once. For the formation of PNDs, MSP1E3D1 was combined with the polymer at a MSP:copolymer molar ratio of 1:10, and the mix was incubated for 1 h at 4 °C with gentle rotation. For the production of MsbA-loaded PNDs, we used a MSP:MsbA molar ratio of 6:1. After incubation of MsbA with the copolymer at 4 °C, with gentle rotation for 10 min, MSP (without poly-His tag) was added. Self-assembly of PNDs was initiated by reducing the concentration of detergent by a 20-fold dilution with the same buffer, but without OG, and the mix was incubated overnight at 4 °C, with gentle rotation, with Ni-NTA beads previously washed with the same buffer. The sample was then loaded onto a column, washed with 3 column volumes of 200 mM NaCl, 20 mM Tris/HCl, pH 7.5, with 5 mM imidazole, followed by 3 column volumes of the same buffer, but with the addition of 0.05% DDM. The PNDs were eluted using 3 column volumes of 200 mM NaCl, 20 mM Tris/HCl, pH 7.5, and 300 mM imidazole, without DDM. The presence of polymer in the elution fractions was determined by the absorbance at 259 nm (A_259_) and that of MSP and MsbA by staining 16% gels (SDS-PAGE) with Instant Blue. The samples were analyzed by SEC using a PL Aquagel-OH 50 column (Agilent Technologies) equilibrated with 200 mM NaCl, 20 mM Tris/HCl, pH 7.5. The flow rate was set at 0.5 ml/min and 1-ml fractions containing PNDs were collected for the studies. The use of dilution to initiate the formation of PNDs was chosen because the copolymer was adsorbed by the Bio-Beads. The addition of DDM during the second wash was an effective and simple way to remove copolymer associated with PNDs, which accounted for a broader size distribution and apparently larger PNDs. DDM was not present after the second wash.

### Estimation of nanoparticle size and size distribution by DLS

DLS experiments were performed at 22 °C on a Zetasizer Nano ZSP (Malvern Instruments), using 40-μl microcuvettes. In general, the samples were centrifuged at 250,000 g for 20 min and the supernatant was used for the DLS measurements. To follow stability over time, the samples were centrifuged only at t = 0. For each sample, measurements were repeated at least 3 times, with each being a 15-scan average (each ~15-s long). Size-intensity distributions were generated using the Zetasizer software version 7.11, and were analyzed using the protein analysis distribution.

### Electron microscopy and image processing of polymersomes and PNDs

The morphology of polymersomes was characterized on a Hitachi H-8100 electron microscope equipped with an AMT digital side mount camera and operated at an accelerating voltage of 75 kV. The polymersomes were stained with 1% uranyl acetate on the TEM grid immediately before taking measurements. To observe PNDs serial dilutions of the sample were stained with uranyl formate as described^64^, using sample buffer (200 mM NaCl, 20 mM Tris/HCl pH 7.5) instead of water for the washes. Specimens were then imaged in a Tecnai 12 electron microscope (FEI Company, Hillsboro, OR) equipped with a Lab6 electron source operated at 120 kV. Micrographs were automatically collected under low-dose conditions using EPU (FEI Company, Hillsboro, OR) at a nominal magnification of 67,000 X. Under-focused images (1 to 3 μm) were recorded on a US4000 CCD camera (Gatan, Pleasanton, CA) with a pixel size at the specimen level of 1.77 Å. The contrast transfer function (CTF) of the images was determined using ctffind^65^. Images were selected based on the following criteria: visual assessment of particle dispersion, quality of stain and background, low astigmatism, and amplitude of signal and correlation with the expected CTF in the frequency range of 50 to 10 Å. All further processing was performed within the framework of EMAN 2.12.66 Particles were extracted with a box size of 150 pixels, CTF corrected and pooled in a set. Reference free classification of down-sampled and low-pass filtered (16 Å) images was used to eliminate “bad” particles and false positives, resulting in a “cleaned” dataset. This set was subject to a second round of classification.

### Data presentation and statistics

Data are shown as means ± SEM, and statistical comparisons were performed by the Student’s t test for unpaired data, or one-way analysis of variance, as appropriate. P < 0.05 in a two-tail analysis was considered significant. The number of experiments (n) corresponds to independent measurements from at least three different preparations.

### Data availability

The datasets generated during and/or analyzed during the current study are available from the corresponding authors on reasonable request.

## Electronic supplementary material

Supplementary Information
