# Supplementary material for: Polymer Nanodiscs: Discoidal Amphiphilic Block Copolymer Membranes as a New Platform for Membrane Proteins
Source: Sci Rep. 2017 Nov 9;7:15227. doi: 10.1038/s41598-017-15151-9 (PMC5680229; doi:10.1038/s41598-017-15151-9)
Supplement: Supplementary file 1 — Supplementary Information [file 41598_2017_15151_MOESM1_ESM.pdf]

SUPPLEMENTARY INFORMATION

Polymer nanodiscs: Discoidal amphiphilic block copolymer membranes as a new platform for membrane proteins

Mariana C Fiori[1*], Yunjiang Jiang[1*], Wan Zheng[1], Miguel Anzaldua[2], Mario J Borgnia[3], Guillermo A Altenberg[1] & Hongjun Liang[1]

[1]Department of Cell Physiology and Molecular Biophysics, and Center for Membrane Protein Research, Texas Tech University Health Sciences Center, Lubbock, Texas, USA. [2]Department of Chemical Engineering, Texas Tech University, Lubbock, Texas 79409, USA. [3]Laboratory of Cell Biology, Center for Cancer Research, National Cancer Institute, National Institutes of Health, Bethesda, Maryland

MATERIALS AND METHODS

MATERIALS

The 4-vinylpyridine (4VP; inhibited with 100 ppm of hydroquinone) was purchased from Sigma Aldrich, and purified by passing through a basic Al_2O_3 column before use. The 1,1′-azobis(cyclohexanecarbonitrile) (ACHN) and azobisisobutyronitrile (AIBN), also purchased from Sigma Aldrich, were recrystallized from methanol twice before use. Hydrogenated hydroxyl-terminated polybutadiene (Krasol® HLBH-P 2000 (HPBD-$(OH)_2$); M_n ~2,100 Da as indicated by the manufacturer) was a gift from Cray Valley USA, and was used as received. Methanesulfonyl chloride, N,N'-dicyclohexylcarbodiimide (DCC), 4-dimethylamino pyridine (DMAP), 1-ethylpiperidinium hypophosphite (EPHP) and methyl iodide were purchased from Sigma Aldrich, and were used as received. *S*-1-dodecyl-S'-(α,α'-dimethyl-α''-acetic acid)trithiocarbonate (DATC) was synthesized according literature[1]. All other chemicals and solvents were obtained from Aldrich and were used as received, except for those anhydrous solvents that were generated using the SPBT-1 Bench Top Solvent Purification System (LC Technology Solutions, Salisbury, MA).

METHODS

The chemical structures of polymers were characterized by ^{1}H NMR (Jeol 400 MHz liquid-state NMR spectrometer), and the polymer size distribution was assessed by size-exclusion chromatography (SEC). SEC was performed on an Agilent 1260 HPLC system equipped with Wyatt Optilab T-rEX refractive index and a Wyatt MiniDAWN TREOS multi-angle light scattering detectors, using an Agilent PLgel 5 μm MIXED-D column (300 x 7.5 mm). The system was equilibrated with a mixture of DMF/THF = 1/3 (v/v) and run at 0.5 ml/min, at 20°C.

Synthesis of the amphiphilic block copolymer HPBD-*b*-$(P4MVP)_2$

Synthesis of $HPBD_{2k}$-$(NH_2)_2$. We first converted the HPBD-$(OH)_2$ into HPBD-$(NH_2)_2$ following a previously reported method, as illustrated in **Fig. 1**, for the preparation of amide-bonded HPBD-*b*-$(P4MVP)_2$ that forms stable

polymersomes[2]. Briefly, methanesulfonyl chloride (4.33 g, 37.8 mmol) and 20 ml anhydrous THF were added to a 200-ml round-bottom flask, and the mixture was placed in an ice bath. To this, a mixture of triethylamine (3.82 g, 37.8 mmol) and HPBD-$(OH)_2$ (10.0 g, 9.50 mmol -OH) in 80 ml anhydrous THF was added dropwise. After 24 h the reaction solution was filtered to remove the insoluble triethylamine hydrochloride, and then precipitated into 10-fold methanol twice. The product HPBD-$(OMs)_2$ was collected by centrifugation (6,000 g for 5 min) and dried under vacuum. The purified HPBD-$(OMs)_2$ (5.0 g, 2.2 mmol) was subsequently dissolved in 60 ml THF and transferred into a Teflon container. After addition of 15 ml of a 28% ammonia aqueous solution, under vigorously stirring, the lid was tightly sealed and the mixture was stirred at 70°C for three days. Ammonia was then allowed to evaporate by air flow in a fume hood. NaOH was then added dropwise from a 5 M solution until the pH reached 13, and the mixture was stirred for 1 h. The concentrated solution was precipitated into 10-fold of methanol, the solid was re-dissolved by dichloromethane, and washed with Millipore water three times. After dehydration with anhydrous $MgSO_4$, the solution was concentrated and precipitated into methanol twice. The product was dried under vacuum and characterized by ^1H NMR.

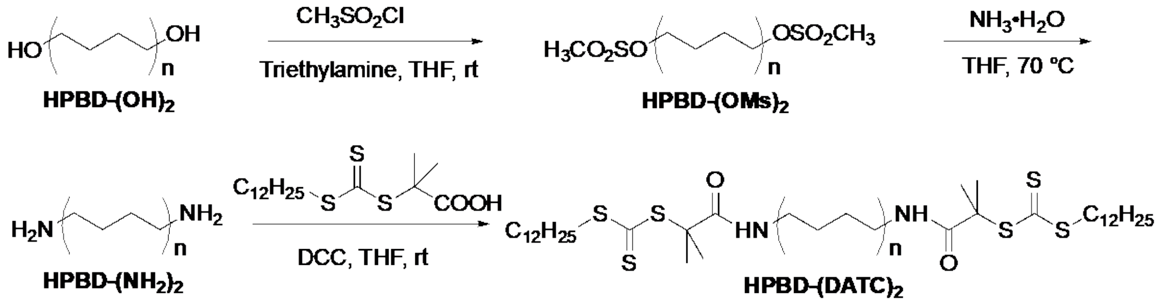

Fig. 1. Synthesis of HPBD-$(NH_2)_2$ and HPBD-$(DATC)_2$

Synthesis of macro-chain transfer agent (macro-CTA) HPBD-$(DATC)_2$. In order to synthesize HPBD-*b*-Poly(1-methyl-4-vinylpyridine)$_2$, *i.e.*, HPBD-*b*-$(P4MVP)_2$ *via* reversible addition-fragmentation chain transfer (RAFT) polymerization, we first prepared macro-CTA HPBD-$(DATC)_2$ by reacting HPBD-$(NH_2)_2$ with DATC *via* DCC coupled amide formation, as shown in **Fig. 1**. Briefly, DCC (2.06 g, 10 mmol) and DATC (3.65 g, 10 mmol) were dissolved in 30 ml of anhydrous dichloromethane in a 100-ml flask. HPBD-$(NH_2)_2$ (1.0 g, 0.93 mmol -NH_2) was dissolved in 50 ml anhydrous dichloromethane and added to the flask dropwise. The mixture was stirred at room temperature for two days. After that, the insoluble solid was filtered away and the solution was concentrated in a rotary evaporator and precipitated into methanol three times. The product HPBD-$(DATC)_2$ was collected by centrifugation, dried under vacuum and characterized by ^1H NMR.

Synthesis of the HPBD-*b*-$(P4MVP)_2$ amphiphilic triblock copolymer by RAFT polymerization. The HPBD-*b*-$(P4VP)_2$ triblock copolymer was synthesized by RAFT polymerization with HPBD-$(DATC)_2$ as the macro-CTA and AIBN as initiator, as shown in **Fig. 2**. In a typical run, HPBD-$(DATC)_2$ (0.2 g, 0.13 mmol DTAC), 4VP (0.76 g, 7.2 mmol) and AIBN (3.0 mg, 0.018 mmol) were dissolved in 1.7 ml THF in a 10-ml Schlenk flask equipped with a magnetic stir bar. After degassing by three freeze-pump-thaw cycles, the flask was immersed in a 60°C-oil bath. After a predetermined time, the mixture was quenched by liquid nitrogen and precipitated into 10-fold hexanes twice. The product was collected by centrifugation, dried under vacuum and characterized by ^1H NMR.

The hydrocarbon tail of the macro-CTA was subsequently removed by a reduction reaction, with 1-ethylpiperidinium hypophosphite (EPHP) as the reducing agent. In a typical run, HPBD-*b*-$(P4VP_{28}\text{-CTA})_2$ (0.3 g, 0.034 mmol), EPHP (36 mg, 0.2 mmol) and ACHN (9.8 mg, 0.04 mmol) were dissolved in 6 ml DMF in a 10-ml Schlenk flask equipped with a magnetic stir bar. The mixture was degassed by three freeze-pump-thaw cycles and the flask was immersed in a 110°C-oil bath for 4 h. The solution was then precipitated into an excess of diethyl ether. The precipitate was re-dissolved by dichloromethane, and washed with Millipore water three times. After dehydration with anhydrous $MgSO_4$, the solution was concentrated and precipitated into hexanes.

To obtain the amphiphilic triblock copolymer, HPBD-*b*-$(P4VP_{28})_2$ was allowed to react with an excess of iodomethane in DMF at 45°C for 24 h. The mixture was precipitated in 10-fold diethyl ether, and the product was dried in a vacuum oven overnight and characterized by ^1H NMR.

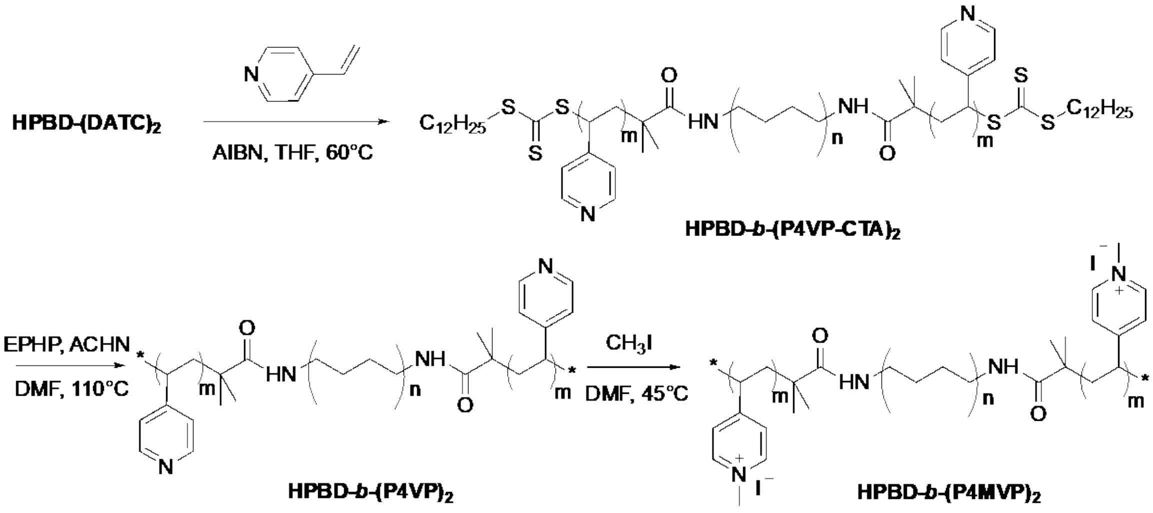

Fig. 2. Synthesis of the amphiphilic triblock copolymer HPBD-*b*-$(P4MVP)_2$ by RAFT polymerization.

Characterization

Synthesis of HPBD-$(NH_2)_2$ and HPBD$_2$-(DATC)$_2$. HPBD-$(OMs)_2$ was first synthesized in order to convert HPBD-$(OH)_2$ to HPBD-$(NH_2)_2$. The structures of HPBD-$(OH)_2$, HPBD-$(OMs)_2$, and HPBD-$(NH_2)_2$ were characterized by ^{1}H NMR, as shown in **Supplementary Fig. 3 A-C**, respectively. For HPBD-$(OH)_2$, both terminal hydroxyl groups were assumed to be bonded with -CH_2-. When the number of this methylene proton (**a, Supplementary Fig. 3A**) was set to 4, there were 328 protons from the backbone. Since each monomer has 8 protons (**b, Supplementary Fig. 3A**) and a formula weight of 56 Da, the actual molecular weight of HPBD-$(OH)_2$ was calculated to be 2,300 Da (M_n), which is slightly larger than the molecular weight (M_n = 2,100 Da) reported by the manufacturer. This NMR-derived molecular weight is in good agreement with that obtained by SEC-MALS measurements, which reports the absolute molecular weight. For HPBD-$(OH)_2$, the MALS analysis reported a M_w = 2,540 and M_n = 2,230 Da, with a polydispersity index (PDI) of 1.14 (**Fig. 2** in the paper). Consequently, we used the 328 protons to account for all protons in the backbone of HPBD when we calculated the composition of other polymer products derived from HPBD-$(OH)_2$ using NMR.

For HPBD-$(OMs)_2$, when the integration of proton **b** was set to 328, there were 6 protons from the end methyl group (**c**, **Supplementary Fig. 3B**), indicating that nearly 100% of hydroxyl groups have been reacted with methanesulfonyl chloride. After aminolysis of HPBD-$(OMs)_2$ the number of protons immediate next to –NH_2 (proton **a** in **Supplementary Fig. 3C**) changed to 3.81, suggesting that >95% of –OH groups have converted to –NH_2.

The HPBD-$(NH_2)_2$ was then reacted with DATC *via* DCC coupled amide formation and the NMR spectrum of the product is shown in **Supplementary Fig. 3D**. Protons **a** and **b** have overlapping chemical shift and the total number was set to 8, assuming 100% functionalization of –NH_2 to graft DATC. Consequently, a total of 407 protons were calculated for protons **c**, **d**, and **e**, a value slightly larger than the expected number (*i.e.*, 386). The overestimation (*i.e.*, extra 21 protons, ~5% of the total) suggests that a very small portion of –NH_2 groups have not been functionalized with DATC.

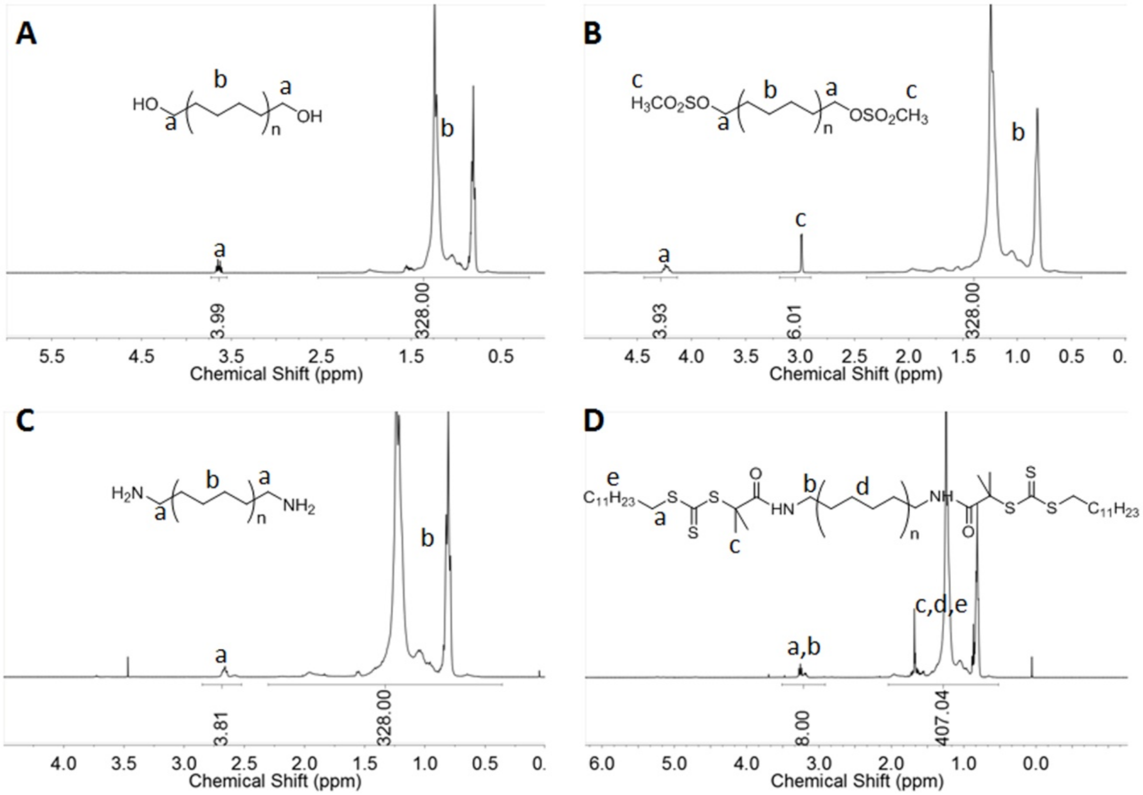

Supplementary Fig. 3. NMR spectra of (A) HPBD-$(OH)_2$, (B) HPBD-$(OMs)_2$, (C) HPBD-$(NH_2)_2$, and (D) HPBD-$(DATC)_2$. All spectra were measured in $CDCl_3$.

Synthesis of the amphiphilic triblock copolymer HPBD-$(P4MVP)_2$. We used HPBD-$(DATC)_2$ as the macro-CTA for the RAFT polymerization of HPBD-*b*-$(P4VP)_2$, and characterized the product by NMR (**Supplementary Fig. 4A**) and SEC-MALS. When proton **c** was set to 4, there were 110 protons from proton **a** and 112 protons from proton **b**, indicating that the average degree of polymerization (DP) of each P4VP block was 28 units. The calculated molecular weight (M_n) of the block copolymer based on this DP was 8,910 Da, which is very similar to the absolute molecular weight measured by SEC-MALS (**Fig. 2** in the paper). The MALS analysis gave a M_w of 10,090 Da, a M_n of 8,690 Da, and a PDI of 1.16. The low PDI suggests that the tri-block copolymer has a focused chain size distribution. The hydrocarbon tail of the CTA was then removed, and the de-alkylated HPBD-*b*-$(P4VP)_2$ was converted to the amphiphilic tri-block copolymer HPBD-$(P4MVP)_2$, Its NMR spectrum is shown in **Supplementary Fig. 4B**. When proton **a** was set to 110, there were 165 protons from proton **c,** suggesting that all 4VP units have been converted to 4MVP.

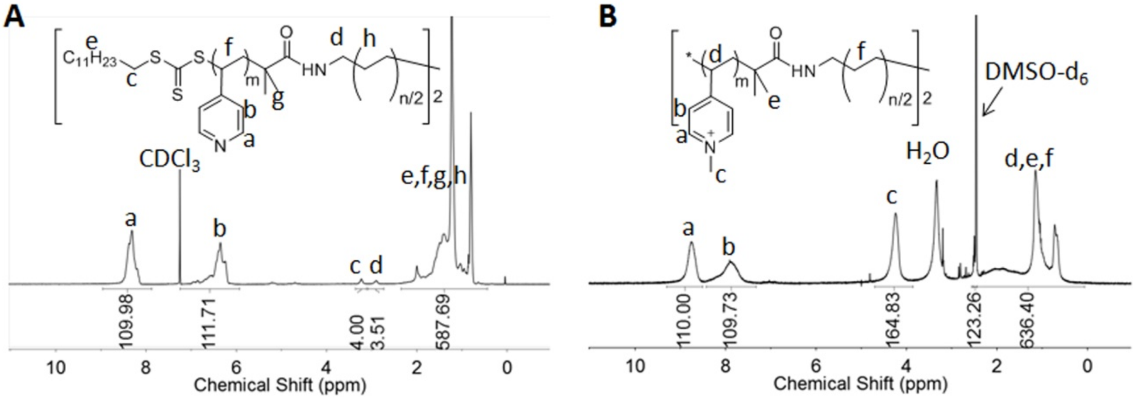

Supplementary Fig. 4. The NMR spectrum of (A) HPBD-*b*-(P4VP)$_2$ and (B) HPBD-*b*-(P4MVP)$_2$.

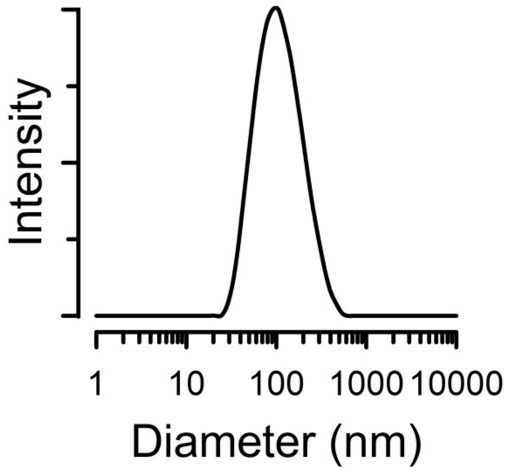

Supplementary Fig. 5. DLS intensity distribution of polymersomes. HPBD-*b*-(P4MVP$_{28}$)$_2$ self-assembled in water showing the formation of polymersomes of different sizes, with a Z-average diameter of ~86 nm.

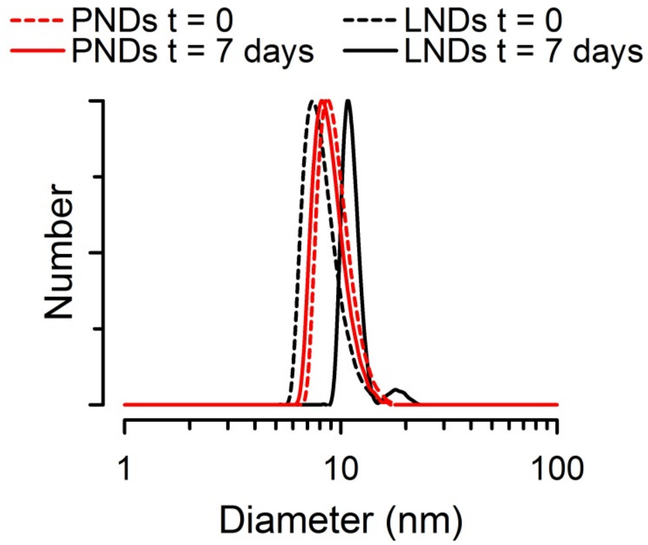

Supplementary Fig. 6. Typical examples illustrating LND and PND hydrodynamic diameter distributions determined by DLS and presented as particle number distributions. Data correspond to the LNDs and PNDs studied at time = 0 and at day 7 of the incubation at 37°C.

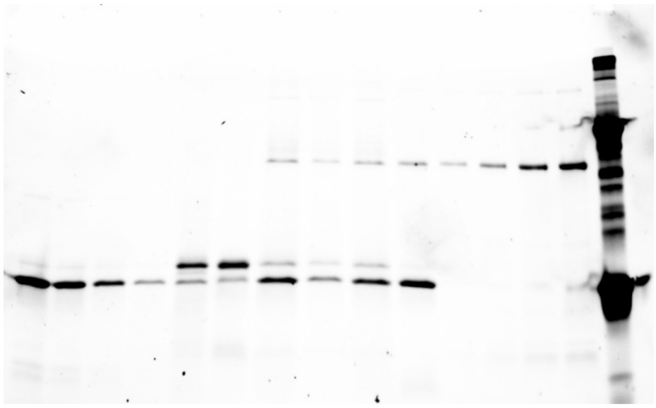

Supplementary Fig. 7. Original gel image used to produce Fig. 3D of the paper. Lanes 3: MSP lane in **Fig. 3D**; lanes 5-6: PNDs and LNDs lanes in **Fig. 3D**, respectively; lanes 9-11: MsbA-PNDs, MsbA-LNDs and MsbA lanes in **Fig. 3D**, respectively.

Preparation of polymersomes. Polymersomes were formed by dissolving HPBD-*b*-(P4MVP)$_2$ in 3/1 (vol. ratio) of DMSO/THF and dialyzed in Millipore water for two days. Their morphology and size distribution are presented in the paper.

References

1. Lai, J.T., Filla, D. & Shea, R. Functional polymers from novel carboxyl-terminated trithiocarbonates as highly efficient RAFT agents. *Macromolecules* **35,** 35, 6754-6756 (2002).
2. Chawla, U. et al. A usual G-protein-coupled receptor in unusual membranes. *Angew Chem Int Ed* **55,** 55, 588-592 (2016).
